# *Chimonanthus nitens* var. *salicifolius* Aqueous Extract Protects against 5-Fluorouracil Induced Gastrointestinal Mucositis in a Mouse Model

**DOI:** 10.1155/2013/789263

**Published:** 2013-12-03

**Authors:** Zhenze Liu, Jun Xi, Sven Schröder, Weigang Wang, Tianpei Xie, Zhugang Wang, Shisan Bao, Jian Fei

**Affiliations:** ^1^School of Life Science and Technology, Tongji University, Shanghai 200092, China; ^2^The Sino-Australia Joint Laboratory, Lishui Institute of Traditional Chinese Medicine, Tongji University, Lishui 323000, China; ^3^Shanghai Research Centre for Model Organisms, Shanghai 201203, China; ^4^HanseMerkur Centre for Traditional Chinese Medicine at the University Medical Centre Hamburg-Eppendorf, Haus Ost 55, UKE Campus, Martinistraße 52, 20246 Hamburg, Germany; ^5^Shanghai Standard Biotech Co., Ltd., Shanghai 201203, China; ^6^Discipline of Pathology, Bosch Institute and School of Medical Sciences, University of Sydney, NSW 2006, Australia

## Abstract

Gastrointestinal mucositis is a major side effect of chemotherapy, leading to life quality reduction in patients and interrupting the therapy of cancer. *Chimonanthus nitens* var. *salicifolius* (CS) is a traditional Chinese herb for enteral disease. Considering the protective effect of CS on intestine, we hypothesize that the aqueous extract of CS could be benefcial to gastrointestinal mucositis. To verify this, a mouse mucositis model was induced by 5-Fluorouracil (5-Fu). Male Balb/C mice were treated with CS aqueous extract (5, 10, and 20 g/kg) or loperamide (0.2 mg/kg) intragastrically for 11 days, and the severity of mucositis was evaluated. Furthermore, the chemical compounds of CS aqueous extract were also analysed by high-performance liquid chromatography (HPLC). Our results demonstrated that CS aqueous extract improved mice body weight, diarrhoea, and faecal blood, maintained the liver function and intestinal length, alleviated villus shortening, and suppressed the apoptosis and inflammation in small intestine. We concluded that CS could protect mice against 5-Fu induced mucositis by inhibiting apoptosis and inflammation, and this protective effect might be associated with the 3 flavonoids (rutin, quercetin, and kaempferol) identified in CS aqueous extract.

## 1. Introduction

*Chimonanthus nitens* var. *salicifolius* (also named *Chimonanthus salicifolius*, abbreviation CS) belongs to Calycanthaceae family and is considered as a unique plant species in China. This plant is generally distributed in the mountain areas of southeast China and it is a semievergreen shrub with solitary and small yellowish flowers. The leaf blade of CS is linear-lanceolate or oblong-lanceolate and has bristle on veins and margin [1]. It is reported that leaves of CS are rich in protein, crude fat, fibers, minerals, vitamin B_2_, and C [2]. Traditionally, the leaves and branchlets of CS are considered as herbs for treating common cold, influenza, dyspepsia, gastritis, enteral disease, and insects bites in part of China [3, 4]. Dry leaves of CS are also boiled as tea by the indigenous people, and they are thought to have a protective effect on gastrointestinal track [4].

Most chemotherapeutic agents target rapidly dividing cells indiscriminately, either malignant or healthy, including intestinal basal stem cells in crypts [5, 6]. Mucositis is the resultant damage to gut induced by chemotherapy, and this side effect limits its clinical applications [7]. Patients with gastrointestinal mucositis usually show abnormal pain, malabsorption, and a range of gastrointestinal symptoms, like vomiting, diarrhoea, ulceration, and bleeding [8–11]. Gastrointestinal mucositis has been reported to occur in 40% of patients receiving standard dose and almost in all patients receiving high dose chemotherapy [12], thus represents a significant clinical and economic burden in oncology [13].

The chemotherapy induced gastrointestinal mucositis involves not only the direct injury to intestinal basal stem cells, but also a consequence of complex biological events, such as reactive oxygen species (ROS) generation, immune cells infiltration, and proinflammatory cytokines oversecretion [14]. Therefore, drugs with antiapoptosis, antioxidative, or anti-inflammatory properties were thought to benefit mucositis patients [15]. Over these few decades, a variety of strategies have been developed for mucositis cure; however, few of these approaches have shown sufficient safety and efficacy [16]. With a growing interest in nature medicine, herb is being adopted as a potential strategy for treating this adverse effect of chemotherapy [17, 18]. More recently, CS has been proved to have an antidiarrhoea effect in mice [19]. However, its function in chemotherapy induced gastrointestinal mucositis is unknown.

In the current study, we utilized 5-fluorouracil (5-Fu), a commonly used chemotherapeutic agent, to induce intestinal injury in Balb/C mice, which mimics human chemotherapy induced mucositis. Three different doses of CS aqueous extract were administered to mice for 11 days, and their possible effect was examined by clinical symptoms, blood biochemical tests, intestinal histopathology, and expression analysis of genes relevant to apoptosis, proliferation, and inflammation. In addition, loperamide, which is used for managing chemotherapy induced mild-moderate diarrhoea [20, 21], was chosen as a symptomatic drug to compare the efficacy with CS aqueous extract. Such data may provide useful information for the prevention or treatment of mucositis in patients undergoing chemotherapy.

## 2. Materials and Methods

### 2.1. Animals

Adult (9 weeks) male BALB/c mice, obtained from Shanghai SLAC Laboratory Animal Co., Ltd. (Shanghai, China), were housed under specific pathogen-free environment with food and water *ad libitum*. The environment was maintained at 22°C with a 12 h light/dark cycle. Animal welfare and experimental procedures were carried out strictly in accordance with the guidance for care and use of laboratory animals (National Research Council of USA, 1996) and approved by Institutional Animal Care and Use Committee of Shanghai Research Centre for Model Organisms.

### 2.2. Preparation of CS Aqueous Extract

CS was obtained from *Tongji University*, *Lishui Institute of Traditional Chinese Medicine* (Lishui, Zhejiang, China). In brief, fresh leaves of CS were crushed and dehydrated at room temperature with constant aeration. Dry material (100 g) was macerated in water (0.6 L) for 2 h and boiled for 10 min. Subsequently, the aqueous phase was harvested, filtered, and centrifuged; supernatant was collected and freeze dried using a freeze dryer (Heto Powerdry PL 3000 Thermo, Thermo Fisher Scientific, USA). Then the extract was redissolved with sterile water into 3 different concentrations (0.25, 0.5, and 1 g/mL of crude drug). Mice were administrated intragastrically with CS aqueous extract twice a day by 10 mL/kg body weight (equivalent to 5, 10, and 20 g/kg).

### 2.3. High Performance Liquid Chromatography (HPLC) Analysis

Main compounds in CS aqueous extract (0.1 g/mL) were analysed using HPLC method. The apparatus system (Agilent Technologies, Germany) was equipped with a binary pump, a diode array detector, and an autosampler. An extend-C18 column (150 nm ∗ 4.6 mm, Agilent, Germany) was used for separation and the temperature was maintained at 25°C. 10 *μ*L of sample was injected into the system and the flow rate was set at 1 mL/min. The mobile phase consisted of methanol (B) and 0.1% phosphoric acid in water (A). The linear gradient elution started at 30% (B), changed to 40% (B) after 15 min, and changed to 60% after 35 min. UV-Vis absorption spectra were monitored by diode array detector at 265 nm. For analysis, the standard substances of rutin, quercetin, and kaempferol were used (Sigma-Aldrich, USA). Compounds in aqueous extract of CS were quantified on the basis of their peak areas and calibration curves of the corresponding standard substance.

### 2.4. Mucositis Inducement and Treatment

5-Fu was purchased from XuDong HaiPu Pharmaceutical Co., Ltd (Shanghai, China). Loperamide (from Xi'an Jangsen Pharmaceutical Ltd, China) was employed to compare the efficacy with CS aqueous extract. Mucositis in mice was induced by 3-day 5-FU administration (50 mg/kg, intraperitoneally) on days 5, 6, and 7, while the controls were treated with saline. Loperamide (0.1 mg/kg) or CS aqueous extract (2.5, 5, and 10 g/kg) was given intragastrically from day 1 to 11, twice daily at 9:00 a.m. and 9:00 p.m. The control and 5-Fu groups were treated with vehicle only (normal drinking water).

### 2.5. Tissue Collection and Mucositis Assessment

Disease severity was assessed by monitoring body weight and scoring for diarrhoea and faecal blood. Diarrhoea score was based on the consistency of stools, using the modified parameters as described before [15, 22]: 0, normal; 1, slightly wet; 2, moderate wet; 3, loose; 4, watery stool. The faecal blood was measured by a commercial testing paper (BASO diagnostics Inc., China) with the following scores: 0, normal; 1, slight bleeding; 2, moderate bleeding; 3, severe bleeding; and 4, visible bleeding.

Serum was collected on day 11 for blood biochemical assays. The small and large intestines were harvested and measured for their lengths immediately following mice sacrifice. Then small intestines were flushed with cold phosphate buffered saline. Part of small intestine was fixed in 4% paraformaldehyde overnight for histological processing as described previously [22], and another part was snap frozen in liquid nitrogen for further analysis.

### 2.6. Serum Biochemical Assays

Serum used for blood biochemical assay was prepared and detected immediately using Chemix-180 automated biochemistry analyzer system (Sysmex Corp, Japan). Liver function/damage following 5-Fu challenge was evaluated by measuring the serum level of alanine aminotransferase (ALT) and aspartate aminotransferase (AST), while the renal function/damage was monitored by the urea nitrogen (BUN) and creatinine (CRE).

### 2.7. Histological Assessment

Paraformaldehyde fixed and paraffin embedded small intestine was sectioned at 5 *μ*m for haematoxylin and eosin (H&E) staining. The morphological assessment was performed by measuring the change in small intestinal villus heights and crypts depths using a light microscope (Nikon 90i, Japan) and the image analysis program of Nikon Nis-element. Thirty villus/crypts were measured in longitudinal tissue sections for each mouse, and all assessments were performed by an independent pathologist in a blind fashion. The shortening of villus was evaluated by the average villus-to-crypt ratio.

### 2.8. Immunohistochemistry

Immunohistochemical detection of proliferating cell nuclear antigen (PCNA) was performed on small intestine sections as described previously [23]. In brief, the sections were incubated with a rabbit polyclonal antibody to PCNA (1 : 100, Abcam biotechnology, China) overnight at 4°C. After washing, slides were stained with a biotin-conjugated secondary antibody (1 : 200, Beyotime Institute of Biotechnology, China), followed by avidin-biotin-peroxidase complex (VECTASTAIN ABC kit, Vector) for 1 hour each at 37°C. The diaminobenzidine was used as the immunodetection substrate. Quantification of PCNA immunohistochemical strain was processed by an Image Pro-Plus program.

### 2.9. Gene Expression Analysis

Total RNA was isolated from snap frozen intestine using a TRIzol A+ reagent (Tiangen Biotech, China) according to the instructions from the manufacturer. cDNA was prepared by reverse transcription using a QuantScript RT kit (Tiangen Biotech, China). The gene expressions of caspase3, PCNA, cyclin-D1, tumor necrosis factor alpha (TNF-*α*), interleukin 1 beta (IL-1*β*), IL-6, and IL-12b were quantified by real-time polymerase chain reaction (PCR), using the SuperReal PreMix Plus kit (Tiangen Biotech, China) and a gradient cycler machine (Eppendorf, Hamburg, Germany). The primer sequences were as follows:

 caspase3: 5′-CTGACTGGAAAGCCGAAACTC-3′ (forward), 5′-CGACCCGTCCTTTGAATTTCT-3′ (reverse);
 PCNA: 5′-TTGCACGTATATGCCGAGACC-3′ (forward), 5′-GGTGAACAGGCTCATTCATCTCT-3′ (reverse);
 cyclin-D1: 5′-TGAGCTTGTTCACCAGAAGCAG-3′ (forward), 5′-TGAGCTTGTTCACCAGAAGCAG-3′ (reverse);
 TNF-*α*: 5′-CCTGTAGCCCACGTCGTAG-3′ (forward), 5′-GGGAGTAGACAAGGTACAACCC-3′ (reverse);
 IL-1*β*: 5′-GAAATGCCACCTTTTGACAGTG-3′ (forward), 5′-TGGATGCTCTCATCAGGACAG-3′ (reverse);
 IL-6: 5′-ACAACCACGGCCTTCCCTACT-3′ (forward), 5′-GCCATTGCACAACTCTTTTCTCAT-3′ (reverse);
 IL-12b: 5′-TGGTTTGCCATCGTTTTGCTG-3′ (forward), 5′-ACAGGTGAGGTTCACTGTTTCT-3′ (reverse);
 
*β*-actin: 5′-ATTGCTGACAGGATGCAGAA-3′ (forward), 5′-GCTGATCCACATCTGCTGGAA-3′ (reverse).

The *β*-actin gene is used as the housekeeping gene and the gene expression is presented as 2^−ΔΔCT^.

### 2.10. Statistical Analyses

All data are showed as mean ± SEM. Differences between groups were estimated using one-way or two-way analysis of variance (ANOVA), followed by the Bonferroni posttest analysis. Significant differences were accepted when *P* values <0.05. The statistical analysis was calculated and plotted using GraphPad Prism version 4.0 (GraphPad Software, Canada).

## 3. Results

### 3.1. Chemical Characterisation of CS Aqueous Extract

**Figure 1:**
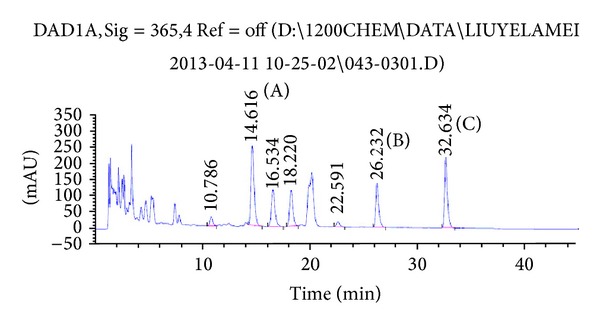
Fingerprint analysis of aqueous extract of CS. HPLC of CS aqueous extract is plotted at 265 nm, using an extend-C18 column and gradient elution with methanol and 0.1% phosphoric acid. The peak identifications are rutin (A), quercetin (B), and kaempferol (C).

CS aqueous extract was prepared and used for chemical analysis by HPLC. The fingerprint is represented in Figure 1. Three flavonoids were identified and quantified. Rutin was the most abundant (0.04088%) in CS aqueous extract, and kaempferol accounted the second major component (0.02892%). Quercetin was also identified (0.01427%).

### 3.2. Effect of CS Aqueous Extract on Body Weight Loss

**Figure 2:**
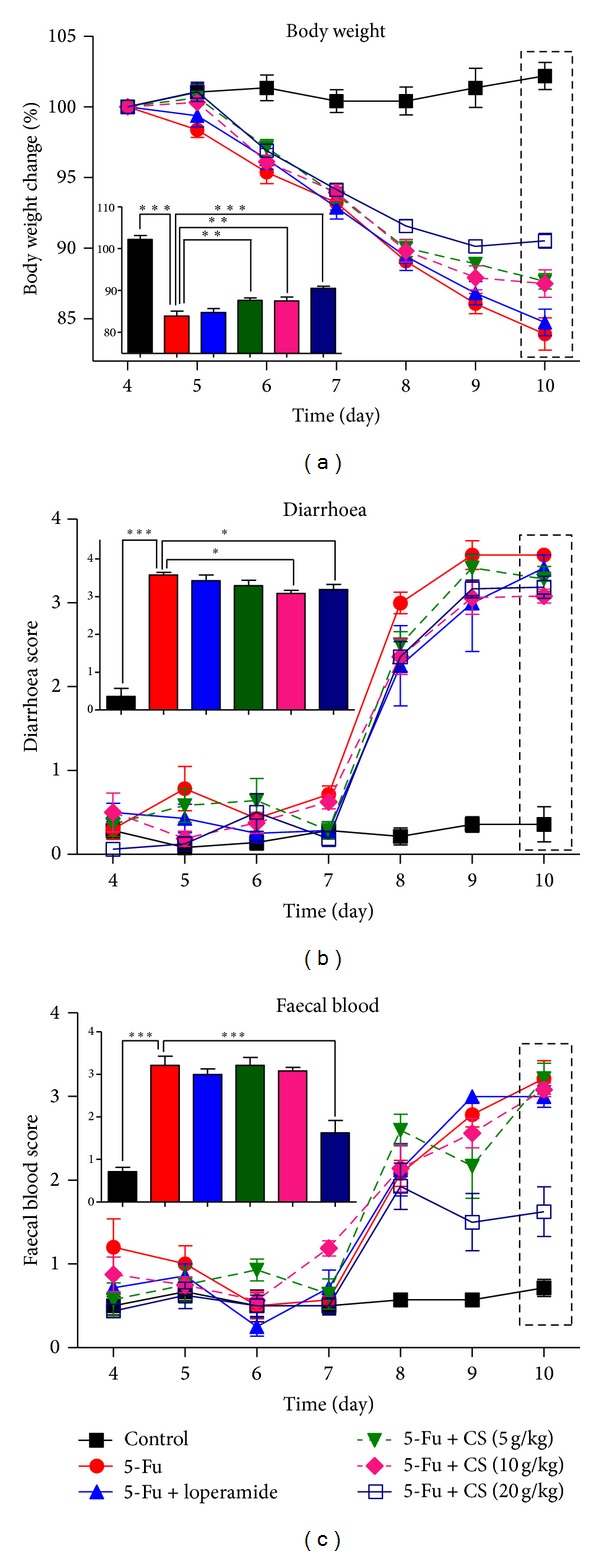
Symptoms develop during mucositis. Bodyweight loss (a), diarrhoea (b), and faecal blood score (c) of mice with vehicle or CS/loperamide treatment were recorded daily. The inset shows data from the day 10, for a better understanding of the relationships among these groups. Each point or bar represents the mean ± SEM (*n* = 8/group); *P < 0.05, **P < 0.01, and ***P < 0.005.

Body weight is an objective criterion for determining the severity of mucositis [6, 15], due to the decrease in food intake and absorption capability. Gradual body weight loss was observed in mice with 5-Fu challenge, reaching ~18% reduction on day 10 (Figure 2(a)). As expected, there was no significant body weight change in the mock treated animals. CS treatment inhibited 5-Fu induced body weight loss at all doses. Significant improved body weight was detected in a dose dependent manner at day 10 (CS 5, 10 g/kg: *P* < 0.01; 20 g/kg: *P* < 0.005). There was no significant difference between 5-Fu and 5-Fu + loperamide treatments (Figure 2(a)).

### 3.3. Effect of CS Aqueous Extract on Diarrhoea and Faecal Blood

Diarrhoea and faecal blood are common gastrointestinal symptoms of mucositis following chemotherapy [24, 25]. There is no abnormality observed in the control group. However, 5-Fu challenge for 3 days caused a sudden elevation of diarrhoea score on day 8 and a progressive increase till day 10. CS (10 and 20 g/kg) and loperamide treatment showed an antidiarrhoea effect at day 8 (diarrhoea score ~2.5, *P* < 0.05). However, only treatment with 10 and 20 g/kg CS reduced the symptom of diarrhoea when it became severe at day 10 (diarrhoea score ~3.5, *P* < 0.05, Figure 2(b)). Consistent with the 5-Fu induced diarrhoea, faecal blood also appeared on day 8, and the faecal blood score worsened daily till sacrifice (Figure 1(C)). Marked improvement was found in 20 g/kg CS aqueous extract treatment group at day 9 (*P* < 0.01) and day 10 (*P* < 0.005). However, such effect was not found in any other treatments (Figure 2(c)).

### 3.4. Effect of CS Aqueous Extract on Liver/Kidney Injury

**Figure 3:**
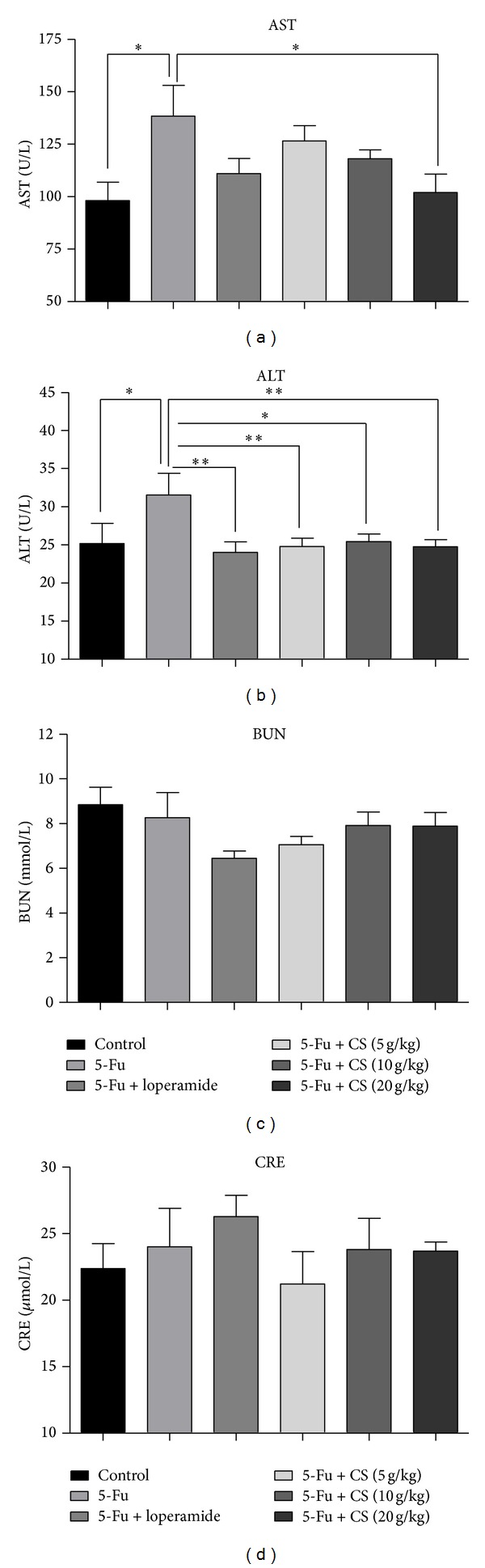
Changes of blood biochemical markers after 5-Fu administration. Serum AST (a), ALT (b), BUN (c), and CRE (d) were determined for monitoring liver and kidney functions. Each bar represents the mean ± SEM (*n* = 8/group); *P < 0.05, and **P < 0.01.

Blood biochemical assay is an easy way to evaluate and diagnose the injury in liver and kidney, which are organs susceptible to the cytotoxicity of chemotherapeutic agents. To determine the protection effect of CS aqueous extract against 5-Fu caused liver and kidney injury, mice blood biochemical indexes were assayed including AST, ALT, BUN, and CRE. 5-Fu challenged mice showed significant increase in AST by ~1.4-fold (*P* < 0.05) and in ALT by 1.3-fold (*P* < 0.05) compared to that of vehicle treated only. CS aqueous extract at all doses (CS: 10 g/kg, *P* < 0.05; 5, 20 g/kg, *P* < 0.01) and loperamide (*P* < 0.01) treatment suppressed the elevation of serum ALT, indicating a protection effect on liver. There was also an attenuation of AST increase in 20 g/kg CS aqueous extract treated mice (*P* < 0.05); however, loperamide did not show such function (Figures 3(a) and 3(b)).

No significant difference in BUN and CRE was found among groups (Figures 3(c) and 3(d)), suggesting that there is no kidney damage following 5-Fu acute challenge.

### 3.5. Effect of CS Aqueous Extract on Intestinal Injury

**Figure 4:**
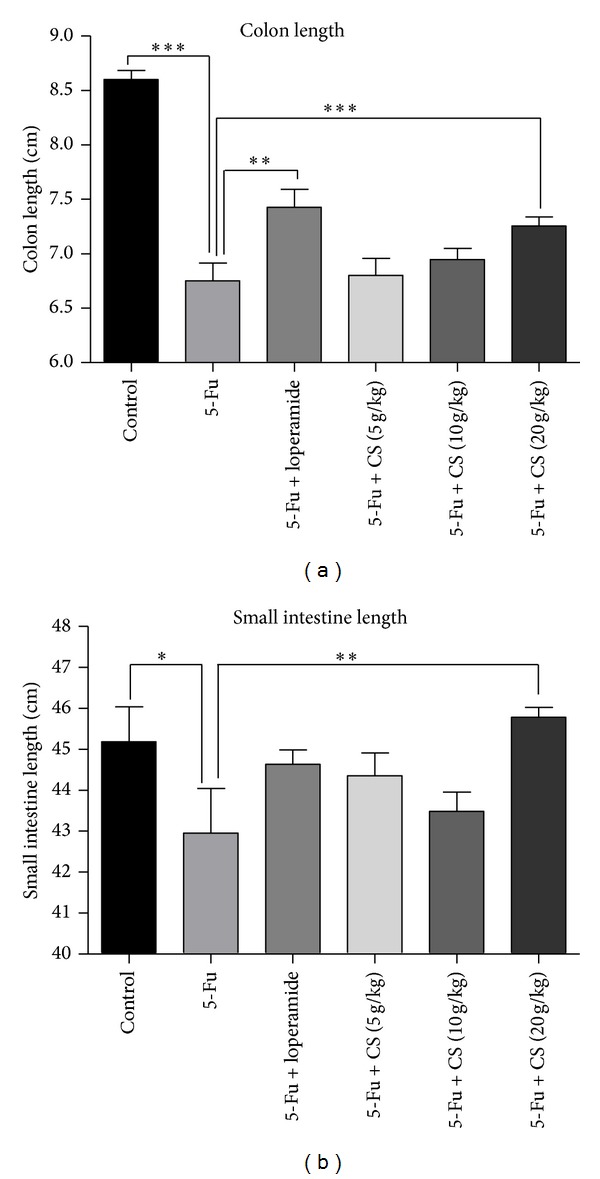
Shortening of colon and small intestine after 5-Fu administration. 4 days after 5-Fu challenge, tissues from different groups were collected, and the shortening of colon (a) and small intestine (b) was detected to evaluate the severity of mucositis. Each bar represents the mean ± SEM (*n* = 8/group); *P < 0.05, **P < 0.01, and ***P < 0.005.

The intestine damage induced by chemotherapy is partially manifested by the reduction of intestinal length [6, 26]. Therefore lengths of large and small intestine with different treatment were determined. The colon length in the 5-Fu treated group was significantly shortened by ~25% compared to that of the mock treated mice (*P* < 0.005), while the reduction of colon length was markedly alleviated by treatment with loperamide (*P* < 0.005) or CS aqueous extract at 20 g/kg (*P* < 0.05). This result suggested that the 5-Fu induced mucositis in colon could be prevented by loperamide or CS aqueous extract (Figure 4(a)). Similar to colon, there was also a shortening in small intestine after 5-Fu challenge (*P* < 0.05), and 20 g/kg CS treatment attenuated the reduction in small intestine length significantly (*P* < 0.01); however loperamide failed to prevent the small intestine shortening (Figure 4(b)).

**Figure 5:**
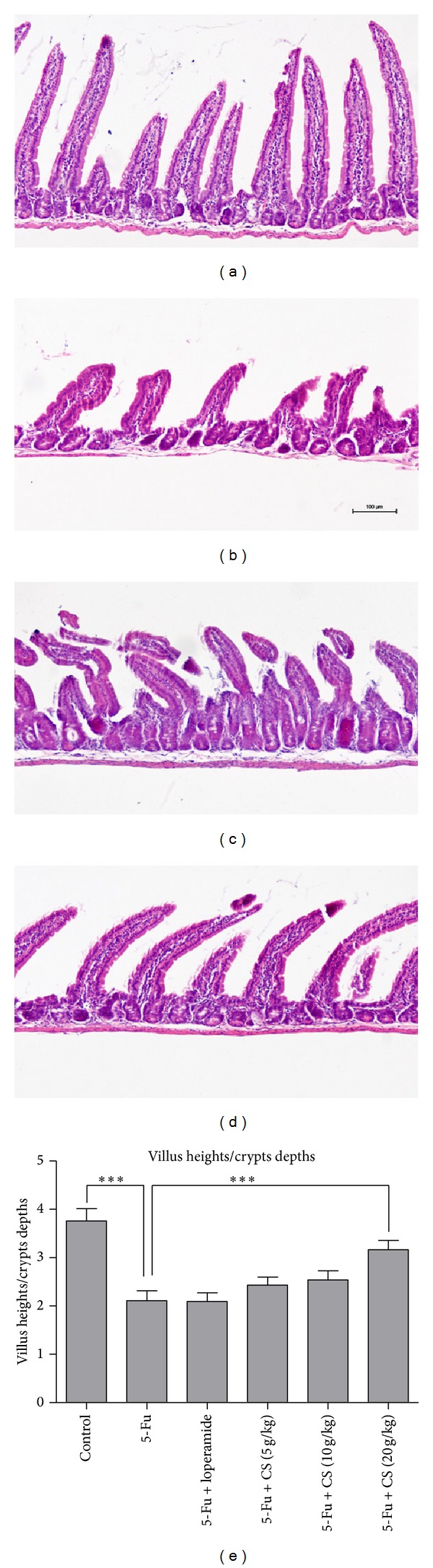
Histological and morphological change of small intestine. Small intestine from control (a), 5-Fu (b), 5-Fu + loperamide (c), and 5-Fu + CS aqueous extract at 20 g/kg (d) was prepared for H&E sections. Morphological assessment was performed using the image analysis program of Nikon Nis-element, and villus shortening was calculated by the average villus-to-crypts ratio (e) to evaluate the mucosal damage. Each bar represents the mean ± SEM (*n* = 8/group); ***P < 0.005.

Furthermore, to evaluate the 5-Fu induced mucosal damage at the microscopic level, small intestine H&E sections were examined (Figures 5(a), 5(b), 5(c), and 5(d)). Compared with the mock treated mice, 5-Fu challenge resulted in villus deformation, loss, and atrophy. Additionally, morphological analysis showed that the ratio of villus heights to crypts depths shortened by ~50% in 5-Fu group than control mice (*P* < 0.005), and treatment with CS aqueous extract at 20 g/kg significantly inhibited the shortening effect induced by 5-Fu (*P* < 0.005). In contrast, loperamide did not prevent the shortening of villus (Figure 5(e)).

### 3.6. Effect of CS Aqueous Extract on Apoptosis and Proliferation

**Figure 6:**
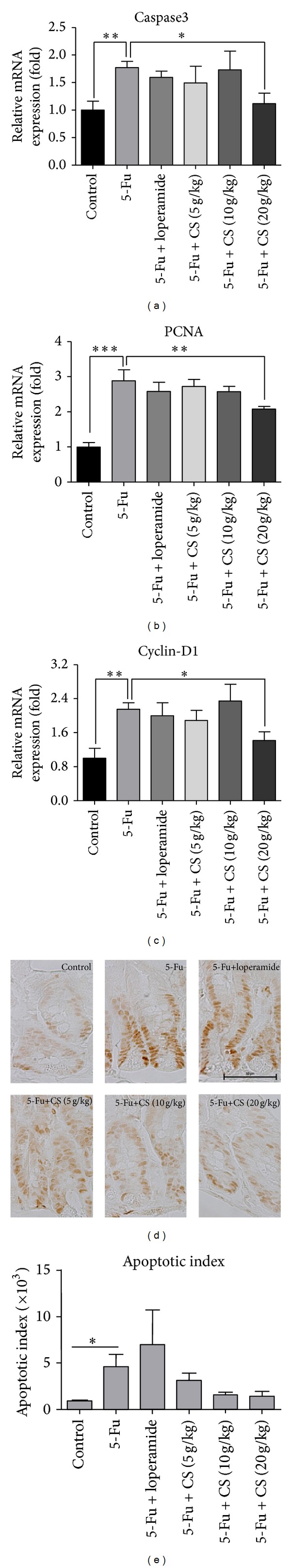
Changes of cell apoptosis and proliferation in mice small intestine. To determine the expression of apoptosis and proliferation relevant genes, expressions of caspase-3 (a), PCNA (b), and cyclin-D1 (c) were quantified by real-time PCR. Moreover, small intestine sections were prepared for immunohistochemical staining of PCNA (d) and quantified for the immunohistochemical density of PCNA positive cells (e). Each bar represents the mean ± SEM (*n* = 8/group); *P < 0.05, **P < 0.01, and ***P < 0.005.

Intestinal basal stem cell is susceptible to the toxicity of chemotherapeutic drugs for its character of rapid turning-over, leading to cell apoptosis in gut tissue and resulting in mucositis. Caspase3 is a member of caspase family and plays a central role in the execution-phase of cell apoptosis [27]. The expression of caspase3 was quantified, and real-time PCR results showed that caspase3 RNA level was increased by 80% in 5-Fu treated group (*P* < 0.01). Such induction was inhibited by CS aqueous extract treatment at 20 g/kg (*P* < 0.05). However no significant inhibition was observed in loperamide treated group (Figure 6(a)).

Cell proliferation is involved in the recovery stage of chemotherapy induced mucositis. As expected, all groups treated with 5-Fu showed an elevation of PCNA expression. The RNA level of 5-Fu mice increased by 80% compared to control mice (*P* < 0.005), and treatment with CS aqueous extract at 20 g/kg reduced the expression of PCNA compared to which with 5-Fu treatment only (*P* < 0.01), suggesting lower proliferation in small intestine (Figure 6(b)). Similar result can be observed in cyclin-D1 (Figure 6(c)). However, no significant effects were observed on PCNA and cyclin-D1 expressions in loperamide treated small intestine (Figures 6(b) and 6(c)). Additionally, the density of PCNA labelling was quantified, using image-Pro plus, as described previously [28]. The data showed that there is a correlation between mRNA and protein for PCNA (Figures 6(d) and 6(e)). These findings showed that CS at 20 g/kg significantly suppressed cellular proliferation, supporting the effect of CS in our current treatment.

### 3.7. Effect of CS Aqueous Extract on Intestinal Inflammation

**Figure 7:**
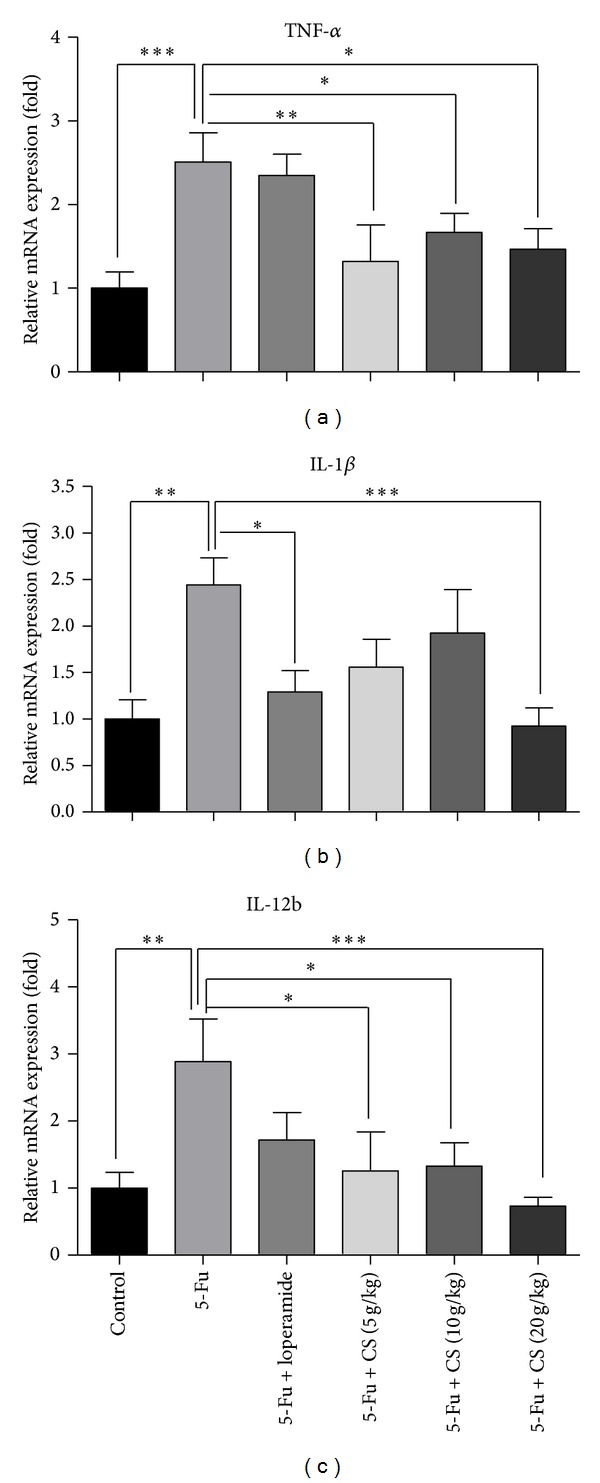
Gene expressions of proinflammatory cytokines in small intestine. To examine the inflammatory status in small intestines, RNA of TNF-*α* (a), IL-1*β* (b), and IL-12b (c) was quantified by real-time PCR. Each bar represents the mean ± SEM (*n* = 8/group); *P < 0.05, **P < 0.01, and ***P < 0.005.

Inflammation is involved in pathogenesis of mucositis, and the proinflammatory cytokines are considered as important factors and potential targets for treatment of mucositis [29, 30]. The gene expressions of cytokines TNF-*α*, IL-1*β*, and IL-12b were quantified. As expected, there were significant increases in TNF-*α* by 1.5- (*P* < 0.005), IL-1*β* by 1.4-, and IL-12b by 1.8-fold (*P* < 0.01) following 5-Fu challenge. Treatment with CS aqueous extract suppressed the elevation of cytokines expression, especially at dose of 20 g/kg (TNF-*α*: *P* < 0.05; IL-1*β*, IL-12b: *P* < 0.005). Loperamide only showed suppression function on IL-1*β* RNA level (*P* < 0.05), but no inhibitory effect was found in TNF-*α* and IL-12b (Figures 7(a), 7(b), and 7(c)).

## 4. Discussions

Gastrointestinal mucositis is a common side effect induced by radio- or chemotherapy, leading to interruptions and delays in tumor treatment [31, 32], and it is one of the primary determinants of morbidity and mortality in oncology [8]. Nowadays there are no broadly effective treatments for mucositis, and the standard managements are limited to pain relief, antidiarrhoeal medication, and infection control [13]. However, couples of strategies are already undergoing investigation, such as keratinocyte growth factor (KGF) [26, 33, 34], insulin-like growth factor-I (IGF-I) [6], IL-11 [35], and glutamine [36]. Traditional herbal medicines also show a potential application in mucositis treatment, and some of them have been reported to be able to benefit the gastrointestinal mucositis complicated with chemotherapy [37–39]. In the present study, we demonstrated the protective effect of CS on 5-Fu induced mice intestinal injury and analysed the fingerprint of CS aqueous extract by HPLC.

Our result showed that CS attenuated the 5-Fu induced body weight loss in a dose dependent manner, indicating improvements in food intake and nutrition. Additionally, CS also alleviated the diarrhoea in mucositis mice. Diarrhoea happened as high as 50~80% in patients that received chemotherapy [25], and might be life threatening when it becomes severe [40]. The chemotherapy induced diarrhoea might be associated with the altered gut motility, impairing water absorption and intestinal microflora [24, 25]. Loperamide, an opioid drug affecting intestinal smooth muscle directly, was commonly used for clinical diarrhoea control [24, 41]. In the present study, loperamide showed an antidiarrhoea effect on moderate symptom at day 8 (score ~2.5) but failed to relieve it when severe episode appeared at day 10 (score ~3.5). This result is consistent with the reported clinical observations [40, 42]. Bleeding is usually accompanied with diarrhoea in patients undergoing chemotherapy [24, 25]. In our results, a marked elevation of faecal blood score was found in 5-Fu challenged mice, and high dose of CS treatment alleviated the stool bleeding. This finding suggested that CS also has a capacity for reducing the ulcerative lesions in gastrointestinal track.

Intestinal shortening is a phenomenon which is described both in chemotherapy induced mucositis [6] and experimental colitis model [22], suggesting destruction or inflammation in intestine. The current study demonstrates that CS aqueous extract treatment (20 g/kg) maintained the length of small and large intestine against 5-Fu induced shortening, while loperamide only showed a protective effect on colon but not on small intestine. Interestingly, compared to colon, the 5-Fu induced shortening in small intestine is quite small, thus might suggest a lighter injury in small intestine than colon. Although there is small significance, the treatment course was rather short (one week) and there might be other factors that contribute to such difference, which will be investigated in our future experiment. Furthermore, histopathological evidence confirmed that there was also a severe mucosal damage in small intestine manifested by villus deformation, loss, and atrophy. CS aqueous extract treatment at 20 g/kg blocked the villus shortening. Such phenomenon can be found in other antimucositis biological agents, such as IGF-I [6] and KGF [26], suggesting that CS aqueous extract might stimulate mucosal growth and optimize the digestive function during chemotherapy. However, loperamide failed to attenuate the atrophy of villus in small intestine.

The pathogenesis of mucositis is complex and multifactorial. Chemotherapeutic agents could interrupt the turning-over of basal stem cells in crypts and induce apoptosis. Our results demonstrated a downregulation of apoptotic gene caspase3 in CS group (20 g/kg) compared with mice treated with 5-Fu only. This data may indicate a prevention effect of CS on apoptosis. In addition, cell apoptosis is just one component of the pathogenesis of chemotherapy induced gut damage. Ceramide signalling, oxidative stress, inflammation, and cell cycling are also implicated in mucositis [43]. The process of mucositis has been described as a 5-phase model, including initiation, primary damage and message generation, signal amplification, ulceration, and healing phase [14, 43]. The direct injury to DNA and generation of ROS are two characterized events in initiation phase [44]. Then a series of transcription factors were prompted to respond to the primary damage, such as nuclear factor *κ*B (NF-*κ*B), Wnt, and p53, [16] which trigger a consequence of molecules, such as proinflammatory cytokines, adhesion molecules, and cyclooxygenase-2 (Cox-2) [43]. TNF-*α* is a crucial cytokine involved in the pathogenesis of mucositis, which amplifies the NF-*κ*B signal and initiates mitogen activated protein kinase (MAPK) pathway [44]. Thus, in line with our findings, CS aqueous extract treatments suppressed the elevation of TNF-*α* induced by 5-Fu challenge. Furthermore, other cytokines, including IL-1*β* and IL-12b, were also inhibited by CS treatment, suggesting an anti-inflammatory effect of CS.

During the stage of healing, epithelium cells proliferate, differentiate to repair the destruction of mucosa, and reestablish the normal local microflora [11, 43]. Interestingly, elevations of PCNA and cyclin-D1 were observed in all 5-Fu treated groups, suggesting a healing process from the experimental mucositis. However, the gene expression is lower in CS treated groups compared with that of vehicle treated only. It remained unclear if the lower level of PCNA and cyclin-D1 in CS treated groups is caused by a direct suppression effect or due to the mild mucositis resulting in less expression of the genes relevant to crypts regeneration.

To analyse the active component, CS aqueous extract is prepared for HPLC detection. Three flavonoids were identified (rutin, quercetin, and kaempferol), and rutin was the most abundant (0.04088%). Rutin is considered as a powerful antioxidant with pharmacological benefits including antitumor, anti-inflammatory, and antidiarrhoeal effects [45, 46]. It has been reported to prevent renal inflammation and apoptosis induced by chemotherapeutic agent cisplatin [45]. The other two compounds, quercetin (0.01427%) and kaempferol (0.02892%), are also described to have protective functions against tumor, oxidative stress, and inflammation [47, 48]. In addition, kaempferol is reported to enhance the intestinal barrier function by expression of tight junction proteins [49]. This information might be useful to explain the biochemical mechanism of CS aqueous extract protecting mice from the development of mucositis.

CS aqueous extract showed effects on inhibiting apoptosis and inflammation in the current experiment, and which seems to play a “protective” role in chemotherapy; however its influence on the antitumor activity of 5-Fu has not been mentioned. The pharmacokinetics of 5-Fu has been well reported in both humans and animals [50, 51]. Furthermore it was demonstrated that there was potential interaction between chemotherapeutic agents and herbs [52], which may result in modification of pharmacokinetics of 5-Fu in the animals with CS treatment. The metabolism and clearance of 5-Fu combined with CS treatment are currently being investigated. Additionally, a (gastrointestinal) tumor model [53] is also needed to evaluate the protective role of CS in gastrointestinal mucositis and its mitigating effect against anticancer chemotherapy. Furthermore, our present work only identified 3 flavonoids. However, there are still a variety of active components present in the extract but without recognization, which might play an important role in CS protective functions. Subsequent studies would further concern in completing the description of various components in CS aqueous extract, and determining the functions of individual compound and mucositis as well as their potential synergistic activity. Thus would provide a better understanding about the mechanism of CS attenuating chemotherapy induced gastrointestinal mucositis.

## 5. Conclusion

The current investigation indicated that the aqueous extract of CS benefits mice against 5-Fu induced gastrointestinal mucositis, attenuating the subsequent body weight loss, diarrhea, and faecal blood, reducing the hepatic injury, and maintaining both intestinal length and villus structure. However, loperamide which is a symptomatic drug for managing mild-moderate diarrhoea induced by chemotherapy only displayed partial protective function. Additionally, this antimucositis activity of CS aqueous extract might be due to inhibiting apoptosis and inflammation in small intestine. Chemical analysis using HPLC identified three major compounds in CS aqueous extract, including rutin, quercetin, and kaempferol. The possible explanation of CS protecting against mucositis might be associated with the characters of these flavonoids, such as antioxidative, anti-inflammatory, antiapoptotic, and antidiarrhoeal effect. Our results suggest that CS is a promising candidate for treatment of gastrointestinal mucositis associated with chemotherapy.

## Abbreviations

CS: *Chimonanthus nitens* var.* salicifolius *
5-Fu: 5-Fluorouracil
ROS: Reactive oxygen species
HPLC: High-performance liquid chromatography
ALT: Alanine aminotransferase
AST: Aspartate aminotransferase
BUN: Urea nitrogen
CRE: Creatinine
H&E: Hematoxylin and eosin
PCR: Polymerase chain reaction
PCNA: Proliferating cell nuclear antigen
TNF: Tumor necrosis factor
IL: Interleukin
ANOVA: Analysis of variance
KGF: Keratinocyte growth factor
IGF-I: Insulin-like growth factor-I
NF-*κ*B: Nuclear factor *κ*B
Cox-2: Cyclooxygenase-2
MAPK: Mitogen activated protein kinase.
